# Deep learning pose estimation for multi-cattle lameness detection

**DOI:** 10.1038/s41598-023-31297-1

**Published:** 2023-03-18

**Authors:** Shaun Barney, Satnam Dlay, Andrew Crowe, Ilias Kyriazakis, Matthew Leach

**Affiliations:** 1 School of Natural and Environmental Science, Newcastle University, Newcastle Upon Tyne, NE1 7RU UK; 2 School of Engineering, Newcastle University, Newcastle Upon Tyne, NE1 7RU UK; 3 Fera Science Ltd, Sand Hutton, York, YO41 1LZ UK; 4 Present Address: Institute for Global Food Security, Queen’s University, Belfast, BT9 5DL UK; 5 Present Address: Comparative Biology Centre, Newcastle University, Newcastle Upon Tyne, NE1 7RU UK

**Keywords:** Behavioural methods, Bioinformatics, Sensors and probes, Sequencing, Animal behaviour, Biomechanics, Image processing, Machine learning, Statistical methods

## Abstract

The objective of this study was to develop a fully automated multiple-cow real-time lameness detection system using a deep learning approach for cattle detection and pose estimation that could be deployed across dairy farms. Utilising computer vision and deep learning, the system can analyse simultaneously both the posture and gait of each cow within a camera field of view to a very high degree of accuracy (94–100%). Twenty-five video sequences containing 250 cows in varying degrees of lameness were recorded and independently scored by three accredited Agriculture and Horticulture Development Board (AHDB) mobility scorers using the AHDB dairy mobility scoring system to provide ground truth lameness data. These observers showed significant inter-observer reliability. Video sequences were broken down into their constituent frames and with a further 500 images downloaded from google, annotated with 15 anatomical points for each animal. A modified Mask-RCNN estimated the pose of each cow to output 5 key-points to determine back arching and 2 key-points to determine head position. Using the SORT (simple, online, and real-time tracking) algorithm, cows were tracked as they move through frames of the video sequence (i.e., in moving animals). All the features were combined using the CatBoost gradient boosting algorithm with accuracy being determined using threefold cross-validation including recursive feature elimination. Precision was assessed using Cohen’s kappa coefficient and assessments of precision and recall. This methodology was applied to cows with varying degrees of lameness (according to accredited scoring, n = 3) and demonstrated that some characteristics directly associated with lameness could be monitored simultaneously. By combining the algorithm results over time, more robust evaluation of individual cow lameness was obtained. The model showed high performance for predicting and matching the ground truth lameness data with the outputs of the algorithm. Overall, threefold lameness detection accuracy of 100% and a lameness severity classification accuracy of 94% respectively was achieved with a high degree of precision (Cohen’s kappa = 0.8782, precision = 0.8650 and recall = 0.9209).

## Introduction

Lameness is one of the most crucial welfare and productivity issues affecting the dairy industry^1^, causing pain and discomfort, resulting in a reduction in milk production and increased risk of early culling^2^. Through early lameness detection and treatment, costs associated with lameness could be reduced. A common method for diagnosing lameness in cattle is through visual observation^3^. This method is both time-consuming and labour intensive, thus constitutes another overlooked cost associated with lameness. The visual locomotion scoring methods commonly used are broadly based on a qualitative assessment of the gait and posture of the cattle^4^. The qualitative nature of the assessment is associated with the high variance between different assessors^5^. The variation between the assessors, and the associated labour costs and time inputs could be removed through a standardized and quantitative automated method.

Existing techniques for automating lameness and pain detection in dairy cattle include leg-load distribution systems^6^, accelerometer-based approaches^7^ and visual inspection through infrared thermography^8^. Various image processing-based techniques are used for lameness detection that monitor different characteristics of the cattle. Pluk et al.^9^ developed an automatic measurement of touch and release angles of the fetlock joint. The system used a synchronized assessment of image processing and pressure data to measure the angle of the metacarpus and metatarsus bones relative to a vertical line in the image. Viazzi et al.^10^ used a system to measure the curvature of the back of an individual that could be used to classify the lameness severity of the cattle. Abdul Jabbar et al.^11^ used an overhead 3-dimensional (3D) depth camera to analyze the cattle gait asymmetry by tracking the hook bones and the spine of the cattle.

These methods are generally very costly as they require the use of specialized equipment^6,9,11^, or are only able to process a single lameness characteristic in each individual cow at any one time, thus reducing their scope in a commercial setting^9,11^. Our approach to addressing early lameness detection in dairy cattle builds upon these existing systems and uses a deep convolutional neural network optimized for instance-based object detection and pose estimation to locate the key points of the cattle associated with detection of lameness. This allows the evaluation of multiple cattle moving in single file through the milking parlor without hindering or slowing down the milking process. Furthermore, our system does not require any specialized equipment, other than a single standard definition video camera. Deep convolutional neural networks are a class of deep neural networks that are predominantly applied to visual based problems such as object classification^12–14^ and object detection^15^. They also include more complex networks that require a hybrid approach to enable object detection, classification, semantic segmentation and pose estimation^16^.

This study aimed to develop a fully automated multiple-cow lameness detection system (i.e., in multiple cows simultaneously) using a deep convolutional neural network for cattle detection and pose estimation. The key points from the pose estimation network are used to monitor the back posture, head position, tracking-up (the cow’s footfall with relation to each other) and movement speed to detect lameness. We focus on the training of the network and the processing of the network output and how these outputs are used as indicators for cattle lameness.

The objective of this study was to develop a prototype, fully automated multiple-cow lameness detection system (i.e., in multiple cows simultaneously) using a deep convolutional neural network for cattle detection and pose estimation that could deployed across dairy farms.

## Results

**Figure 1:**
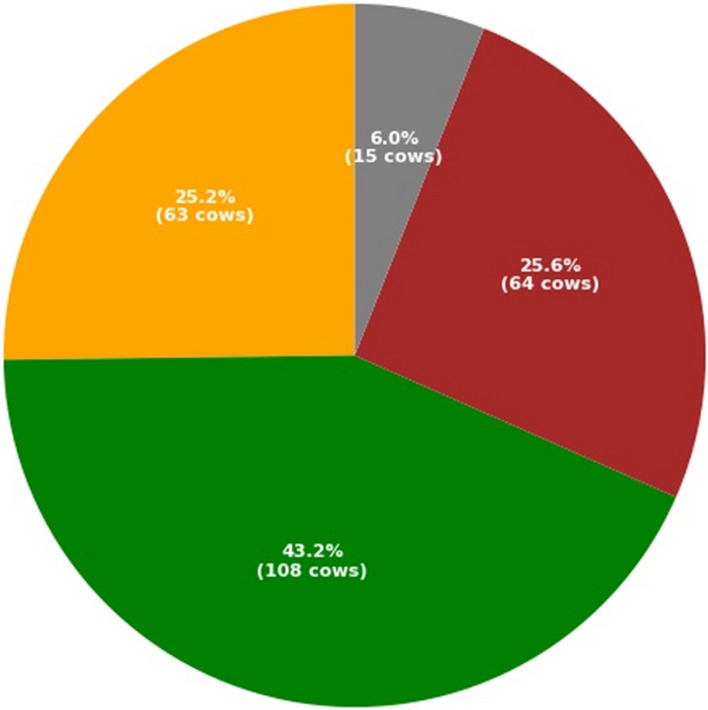
The distribution of lameness scores taken as an average of scores from the three independent registered accredited mobility scorers. Of the total 250 cows, 25.2% of them were assigned a lameness score of 0 (orange), 43.2% with a lameness score of 1 (green), 25.6% with a lameness score of 2 (red) and 6% with a lameness score of 3 (grey).

The entire dataset containing 250 cows were evaluated for lameness based on the assessment of the three independent registered accredited mobility scorers. To ensure consistent behavioural scoring, with the three observers all being accredited mobility scorers, and all scoring the same videos. Kendall’s coefficient of concordance demonstrated significant inter-observer reliability with P < 0.001 and τ = 0.504. Figure 1 shows the balance of the dataset, with 25.2% of the total cows being assigned a lameness score of 0, 43.2% of the cows assigned a lameness score of 1, 25.6% of the cows being assigned a lameness score of 2 and the final 6.0% of the cows being assigned a lameness score of 3.

### Back analysis

In every frame, each cow’s pose was estimated via the Mask-RCNN algorithm and stored in a data frame, with each cow having a unique ID from the tracking algorithm. As the cow moves through frames, the pose of the cows is stored against their unique ID so analysis over time can be achieved. The first analysis compared the RMSE of the back to its associated accredited assigned lameness score (0–3). For each cow, the RMSE was calculated and analysed over time, calculating kurtosis, max value, mean, median, min value, skew, and the standard deviation. Each of these metrics (kurtosis, max value, mean, median, min value, skew, and the standard deviation) were then inspected using Pearson’s correlation against the lameness score for all the 61 cows (results outlined in Table 1).

**Table 1:** Pearson’s correlation coefficients for the root mean squared error of the residuals for the back position against the cow’s lameness score 0–3.

Measurement	Rho^a^
Back RMSE kurtosis	− 0.009^†^
Back RMSE max	0.5163***
Back RMSE mean	0.6125***
Back RMSE median	0.64***
Back RMSE min	0.13^†^
Back RMSE skew	− 0.137^†^
Back RMSE std	0.4118***

^a^The root mean squared errors were calculated from the residuals of key points on the back to the back regression line (outlined in “Back posture analysis”). The indicated probability thus: ^†^*P* < 0*.*10*,* *P < 0*.*05*,* **P < 0*.*01*,* ***P < 0*.*001.

Table 1 shows the median value has the highest correlation with the individual categories of lameness (i.e., 0–3), however the max value, mean and standard deviation are also highly correlated.

Further analysis was undertaken on the back (outlined in “Back area analysis”). Again, for each frame and each cow in the frame, the back area was calculated and stored against the cows' unique ID. For each section of the back the kurtosis, max value, mean, median, min value, skew and the standard deviation were stored to create a feature for that cow to later use in the classification algorithm. As previously stated, each of the metrics was inspected with Pearson’s correlation to see if there was any linear relationship with the lameness score assigned by the accredited scorers (Table 2).

**Table 2:** Pearson’s correlation coefficients for the various areas of the back with relation to the lameness score of the cow 0–3.

Measurement	Rho^a^
Back hip to centre area kurtosis	0.084^†^
Back hip to centre area max	0.628***
Back hip to centre area mean	0.693***
Back hip to centre area median	0.680***
Back hip to centre area min	0.550***
Back hip to centre area skew	− 0.085^†^
Back hip to centre area Std	0.298**
Front shoulder to centre area kurtosis	0.034^†^
Front shoulder to centre area max	0.475***
Front shoulder to centre area median	0.584***
Front shoulder to centre area mean	0.598***
Front shoulder to centre area min	0.439***
Front shoulder to centre area skew	− 0.059^†^
Front shoulder to centre area Std	0.207*
Neck to shoulder area kurtosis	0.013^†^
Neck to shoulder area max	0.337***
Neck to shoulder area mean	0.445***
Neck to shoulder area median	0.409***
Neck to shoulder area min	0.351***
Neck to shoulder area skew	− 0.046^†^
Neck to shoulder area Std	0.260**
Total area kurtosis	0.060^†^
Total area max	0.585***
Total area mean	0.684***
Total area median	0.677***
Total area min	0.534***
Total area skew	− 0.104^†^
Total area Std	0.250*

^a^The individual area calculations are outlined in “Back area analysis”). The indicated probability thus: ^†^*P* < 0*.*10*,* *P < 0*.*05*,* **P < 0*.*01*,* ***P < 0*.*001.

Table 2 indicates many strong correlations between the relative positions of the back key-points and the mobility score, with strongest correlations being associated with key-points located towards the rear of the animal e.g. Back Hip to Centre Area and the total back area metrics.

### Head and neck analysis

For head and nose position, mean position of the head has the strongest correlation with the lameness scores (Table 3). However, the results also show that there is a significant correlation between the lameness score and the minimum and maximum positions of both the head and nose. For the measures of the angle of the neck there is a significant correlation between the standard deviation of the neck angle and the lameness score (Table 4).

**Table 3:** Pearson’s correlation coefficients for the head nose mean squared error with the lameness score 0–3 for all 61 cows.

Measurement	Rho^a^
Head nose mse kurtosis	0.029^†^
Head nose mse max	0.265*
Head nose mse mean	0.348**
Head nose mse min	0.244*
Head nose mse skew	− 0.09^†^
Head nose mse std	0.084^†^

^a^The head nose mean squared error is calculated as the position of the head and nose with relation to the back regression line (outlined in “Back posture analysis” and “Head position analysis”). The indicated probability thus: ^†^*P* < 0*.*10*,* *P < 0*.*05*,* **P < 0*.*01*,* ***P < 0*.*001.

**Table 4:** Displays the Pearson’s correlation coefficients for the angle of the neck with the lameness score 0–3 for all 61 cows.

Measurement	Rho^a^
Neck angle min	0.028^†^
Neck angle max	0.008^†^
Neck angle mean	0.04^†^
Neck angle median	0.05^†^
Neck angle std	− 0.06*
Neck angle kurtosis	0.008^†^
Neck angle skew	− 0.041^†^

^a^The neck angles were calculated as the angle of the neck with relation to the back (outlined in “Neck angle analysis”). The indicated probability thus: ^†^*P* < 0*.*10*,* *P < 0*.*05*,* **P < 0*.*01*,* ***P < 0*.*001.

### CatBoost

**Figure 2:**
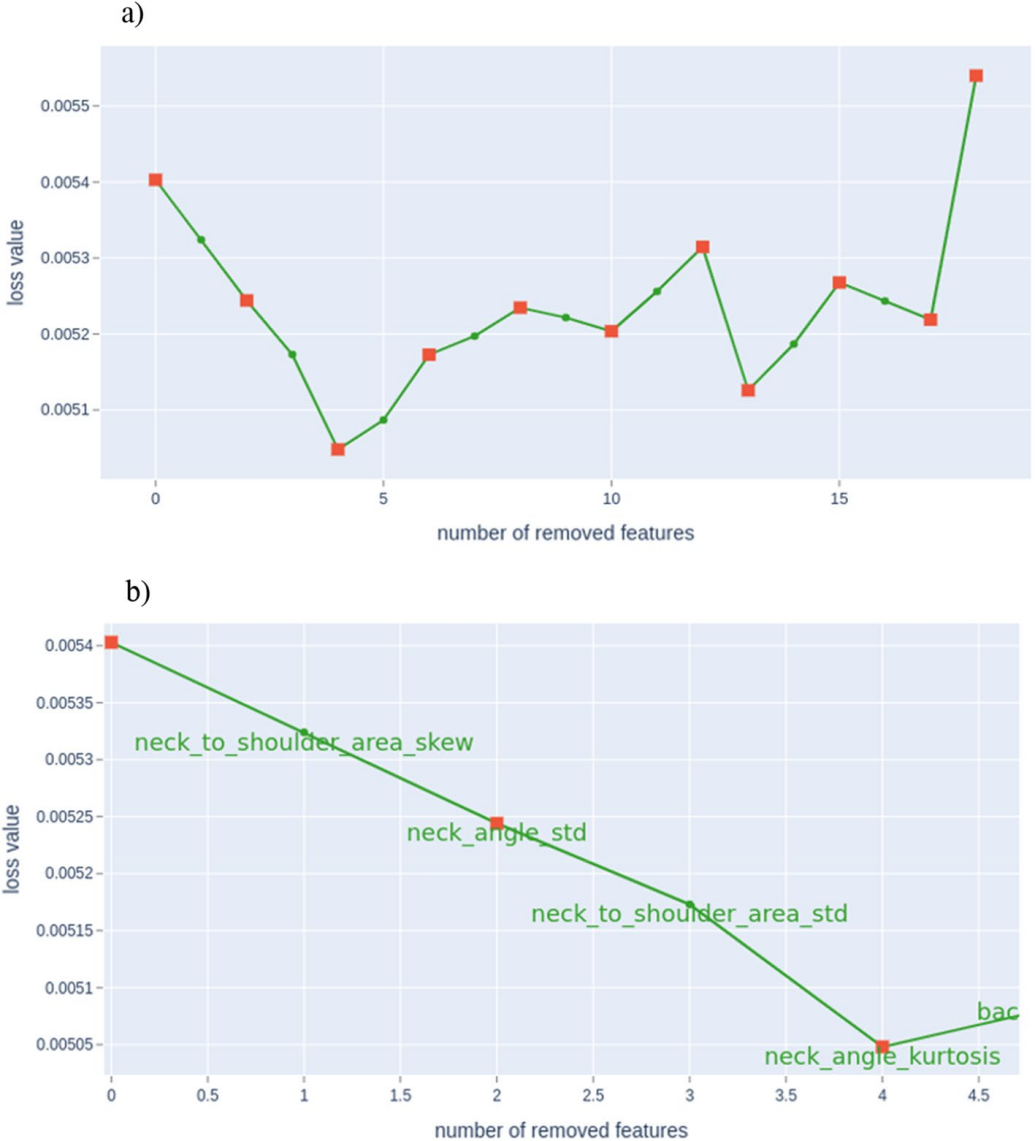
(**a**) The change in error for features removed using the recursive feature elimination algorithm on the Catboost model. The model achieves its lowest error value once 4 features are removed. (**b**) The change in error for the first 4 features removed using the recursive feature elimination algorithm on the Catboost model. The 4 features removed to reduce the error are ‘neck to shoulder area skew’, ‘neck angle standard deviation’, ‘neck to shoulder area standard deviation’ and ‘neck angle kurtosis’.

Using boosting ensemble tree-based machine learning in Python with the Yandex CatBoost library, the features were inspected to determine their normalized and cumulative importance in lameness detection, i.e., against the accredited lameness scores. The absolute value of the feature importance is not as significant as the normalized values which define the most critical features for the lameness classification. This also provides further useful information on the features with zero importance in a tree-based model, as the features with zero importance are not used to split the nodes, therefore, they can be removed without affecting the performance of the model. Figure 2a shows the change in error as the features are recursively removed, with the minimum error being achieved after 4 features were removed. Figure 2b shows a focused plot of the 4 features that were removed to achieve the lowest error—‘neck to shoulder area skew’, ‘neck angle standard deviation’, ‘neck to shoulder area standard deviation’ and ‘neck angle kurtosis’.

To understand which the essential and non-essential features are, we used gradient boosting on each feature averaged over ten training runs to reduce variance. The models were trained using early stopping with a patience of 20 epochs with the validation set to prevent overfitting to the training data.

**Figure 3:**
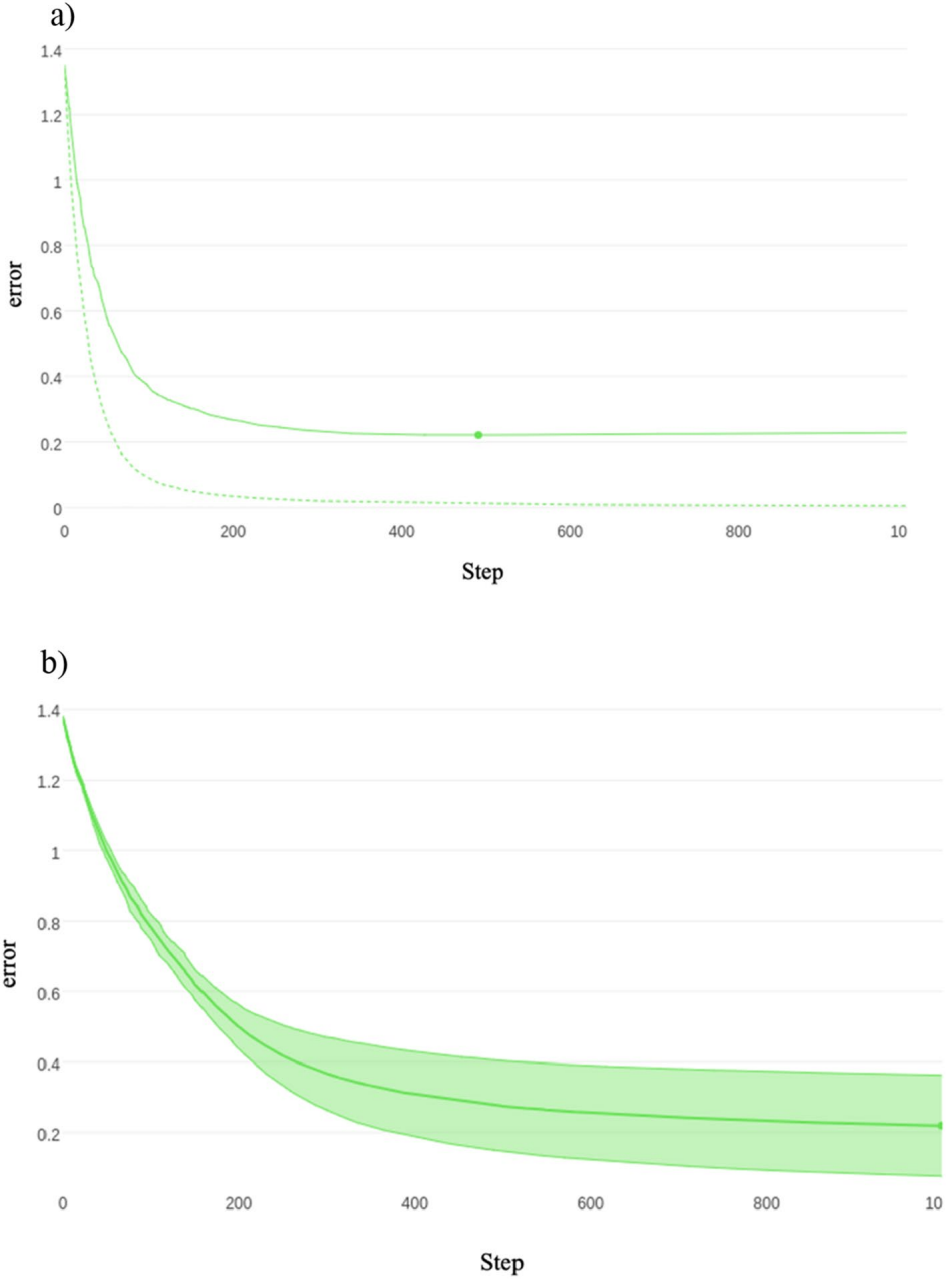
(**a**) Visual representation of the training (shown as the dotted line) and validation (shown as the solid line) losses for the Catboost algorithm on 4 classes. The minimum error of 0.2211 was achieved on step 491, with the training error following a similar curve indicating over fitting has not taken place. (**b**) Visual representation of the validation losses and standard deviation for the Catboost algorithm on 4 classes for threefold cross validation. The average accuracy for the threefold cross validation was 94% ± 0.05.

For the first permutation (classification against 4 assigned mobility categories), using the features outlined in Table 5, the Catboost algorithm was trained to classify each cow to its correct lameness score with a resulting threefold classification accuracy of 94 ± 0.5%, see Fig. 3a for the training and validation curve for a single train and Fig. 3b for the validation graph for the threefold cross validation.

**Table 5:** Normalized and cumulative importance for each of the features where the normalized importance is greater than zero.

Feature	Normalized importance^a^	Cumulative importance^b^
Total back area mean	0.312343	0.312343
Back hip to centre area max	0.115869	0.428212
Back hip to centre area median	0.090680	0.518892
Back RMSE median	0.073048	0.591940
Total back area max	0.037783	0.629723
Head nose MSE std	0.037783	0.667506
Total back area median	0.037783	0.705290
Back RMSE skew	0.037783	0.743073
Front shoulder to centre area min	0.035264	0.778338
Back RMSE std	0.035264	0.813602
Front shoulder to centre area max	0.035264	0.848866
Neck to shoulder area median	0.027708	0.876574
Neck to shoulder area min	0.025189	0.901763
Back RMSE max	0.022670	0.924433
Head nose MSE mean	0.017632	0.942065
Back hip to centre area min	0.017632	0.959698
Neck to shoulder area std	0.010076	0.969773
Front shoulder to centre area mean	0.010076	0.979849
Neck angle kurtosis	0.010076	0.989924
Total back area min	0.007557	0.997481
Head nose MSE min	0.002519	1.000000

^a,b^The importances were calculated using boosting ensemble tree-based machine learning as a factor of how much they contributed to the correct classification for each of the assigned mobility categories (0, 1, 2 or 3).

The Cohen’s kappa coefficient with variance equal-zero test was used to calculate the precision of the classifier across the varying degrees of lameness, see Table 6 for the results with the Cohen’s kappa score of 0.8782. Using the same confusion matrix, we calculate the precision and recall of the model to be 0.8650 and 0.9209 respectively, see Table 6.

For the second permutation (classification against ‘Sound’ [0] or ‘Lame’ [1–3]), using the features outlined in Table 7, the Catboost algorithm was trained as a binary classifier with mobility score 0 as ’Sound’ and mobility scores 1–3 as ’Lame’. The resulting classifier achieved a threefold classification accuracy of 98 ± 0.3%.

The Cohen’s kappa coefficient with variance equal-zero test was used to calculate the precision of the classifier across the varying degrees of lameness, see Table 8 for the results with the Cohen’s kappa score of 0.8403. Using the same confusion matrix, we calculate the precision and recall of the model to be 0.9104 and 0.9388 respectively, see Table 8.

For the third permutation (classification against ‘Little or no lameness’ [0 1] or ‘Clearly lame’ [2 3]), using the features outlined in Table 9, the Catboost algorithm was trained as a binary classifier with assigned mobility scores of 0 and 1 as ‘Little or no lameness’ and assigned mobility scores 2 and 3 as ‘Clearly lame’. The resulting classifier achieved a threefold classification accuracy of 100 ± 0%.

The Cohen’s kappa coefficient with variance equal-zero test was used to calculate the precision of the classifier across the varying degrees of lameness, see Table 10 for the results with the Cohen’s kappa score of 0.8162. Using the same confusion matrix, we calculate the precision and recall of the model to be 0.8963 and 0.9182 respectively, see Table 10.

**Table 6:** Cohen’s kappa coefficient, recall and precision results for the multi-lameness classifiers.

Measurement	Value
Kappa	0.8782
Alpha’s standard error (ASE)	0.0253
95% lower conf limit	0.8287
95% upper conf limit	0.9277
Recall	0.9209
Precision	0.8650

**Table 7:** Normalized and cumulative importance for each of the features where the normalized importance is greater than zero.

Feature	Normalized importance^a^	Cumulative importance^b^
Head nose MSE min	0.206107	0.206107
Neck to shoulder area median	0.099237	0.305344
Front shoulder to centre area min	0.091603	0.396947
Back RMSE kurtosis	0.083969	0.480916
Neck angle std	0.076336	0.557252
Back RMSE std	0.076336	0.633588
Back hip to centre area min	0.068702	0.702290
Total back area max	0.068702	0.770992
Back hip to centre area max	0.053435	0.824427
Front shoulder to centre area max	0.030534	0.854962
Back RMSE max	0.022901	0.877863
Back RMSE skew	0.022901	0.900763
Neck to shoulder area min	0.015267	0.916031
Back RMSE median	0.015267	0.931298
Back hip to centre area mean	0.015267	0.946565
Head nose MSE mean	0.015267	0.961832
Head nose MSE max	0.015267	0.977099
Neck angle mean	0.007634	0.984733
Back hip to center area median	0.007634	0.992366
Back RMSE mean	0.007634	1.000000

^a,b^The importance values were calculated using boosting ensemble tree-based machine learning as a factor of how much they contributed to the correct binary classification where the labels were ‘Sound’ with an assigned mobility score of 0 and ‘Lame’ with assigned mobility scores of 1–3.

**Table 8:** Cohen’s kappa coefficient, recall and precision results for the multi-lameness classifiers.

Measurement	Value
Kappa	0.8403
Alpha’s standard error (ASE)	0.0383
95% lower conf limit	0.7653
95% upper conf limit	0.9154
Recall	0.9388
Precision	0.9104

**Table 9:** Normalized and cumulative importance for each of the features where the normalized importance is greater than zero.

Feature	Normalized importance^a^	Cumulative importance^b^
Back hip to centre area mean	0.369458	0.369458
Total back area mean	0.266010	0.635468
Back RMSE median	0.142857	0.778325
Total back area max	0.137931	0.916256
Total back area median	0.044335	0.960591
Back RMSE std	0.019704	0.980296
Back hip to centre area max	0.009852	0.990148
Back RMSE max	0.0098562	1.000000

^a,b^The importance’s were calculated using boosting ensemble tree-based machine learning as a factor of how much they contributed to the correct binary classification where the labels were ‘little or no lameness’ with assigned mobility scores of 0 and 1 and ‘Clearly lame’ with assigned mobility scores of 2 and 3.

**Table 10:** Cohen’s kappa coefficient, recall and precision results for the multi-lameness classifiers.

Measurement	Value
Kappa	0.8162
Alpha’s standard error (ASE)	0.0393
95% Lower conf limit	0.7391
95% Upper conf limit	0.8933
Recall	0.9182
Precision	0.8963

For the final permutation (classification against ‘No to moderate lameness’ [0–2] or ‘Severely lame’ [3]), using the features outlined in Table 11, the Catboost algorithm was trained as a binary classifier with assigned mobility scores of 0, 1 and 2 as ‘No to moderate lameness’ and a score of 3 as ‘Severely lame’. The resulting classifier achieved a threefold classification accuracy of 97 ± 0.5%.

**Table 11:** Normalized and cumulative importance’s for each of the features with normalized importance greater than zero.

Feature	Normalized importance^a^	Cumulative importance^b^
Back hip to centre area max	0.155172	0.155172
Head nose MSE std	0.120690	0.275862
Neck to shoulder area kurtosis	0.120690	0.396552
Neck to shoulder area min	0.103448	0.500000
Front shoulder to centre area std	0.086207	0.586207
Front shoulder to centre area min	0.086207	0.672414
Total back area kurtosis	0.068966	0.741379
Head nose MSE max	0.051724	0.793103
Neck angle kurtosis	0.034483	0.827586
Neck to shoulder area max	0.034483	0.862069
Back hip to centre area min	0.034483	0.896552
Head nose MSE mean	0.034483	0.931034
Neck to shoulder area std	0.034483	0.965517
Back hip to centre area kurtosis	0.017241	0.982759
Back RMSE mean	0.017241	1.000000

^a,b^The importance’s were calculated using boosting ensemble tree-based machine learning as a factor of how much they contributed to the correct binary classification where the labels were ‘No to moderate lameness’ for assigned mobility scores of 0–2 and ‘Severely lame’ for an assigned mobility score of 3.

The Cohen’s kappa coefficient with variance equal-zero test was used to calculate the precision of the classifier across the varying degrees of lameness, see Table 12 for the results with the Cohen’s kappa score of 0.5020. Using the same confusion matrix, we calculate the precision and recall of the model to be 0.7165 and 0.9283 respectively, see Table 12.

**Table 12:** Cohen’s kappa coefficient, recall and precision results for the multi-lameness classifiers.

Measurement	Value
Kappa	0.5020
Alpha’s standard error (ASE)	0.0819
95% lower conf limit	0.3414
95% upper conf limit	0.6626
Recall	0.9283
Precision	0.7165

### Scorer variability CatBoost classification validation

**Figure 4:**
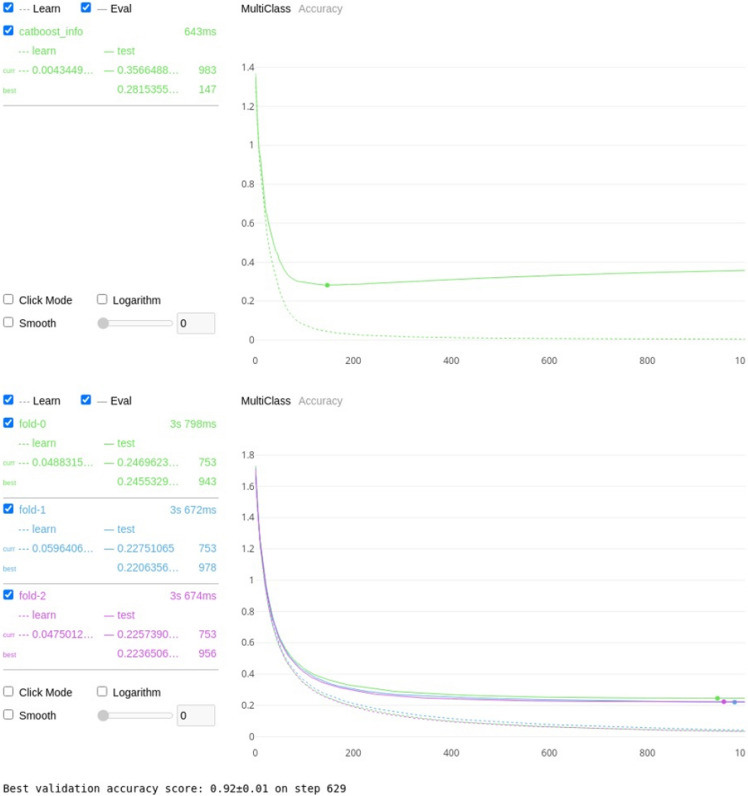
Training and validation plots for scorer 1.

Table 13 shows the results for the CatBoost classification model trained and validated against scorer 1 and Fig. 4 shows the training and validation plots to ensure no overfitting.

**Table 13:** Catboost threefold cross validation accuracy for scorer 1 for each of the 3 lameness score groups: separate degrees of lameness, scores 0 as non-lame with scores 1, 2 and 3 as lame, scores 0 and 1 as non-lame with scores of 2 and 3 as lame, scores 0, 1 and 2 as non-lame and a score of 3 as lame.

Lameness score	Threefold cross validation accuracy (%)
All	92
Lameness ≥ 1	95
Lameness ≥ 2	94
Lameness ≥ 3	98

**Figure 5:**
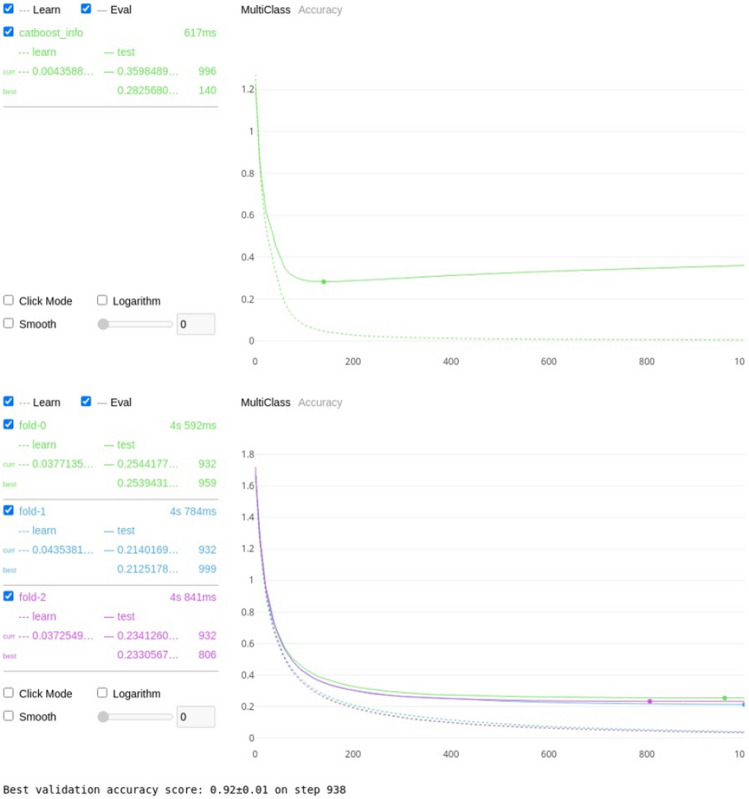
Training and validation plots for scorer 2.

Table 14 shows the results for the CatBoost classification model trained and validated against scorer 2 and Fig. 5 shows the training and validation plots to ensure no overfitting.

**Table 14:** Catboost threefold cross validation accuracy for scorer 2 for each of the 3 lameness score groups: separate degrees of lameness, scores 0 as non-lame with scores 1, 2 and 3 as lame, scores 0 and 1 as non-lame with scores of 2 and 3 as lame, scores 0, 1 and 2 as non-lame and a score of 3 as lame.

Lameness score	Threefold cross validation accuracy (%)
All	92
Lameness ≥ 1	94
Lameness ≥ 2	95
Lameness ≥ 3	98

**Figure 6:**
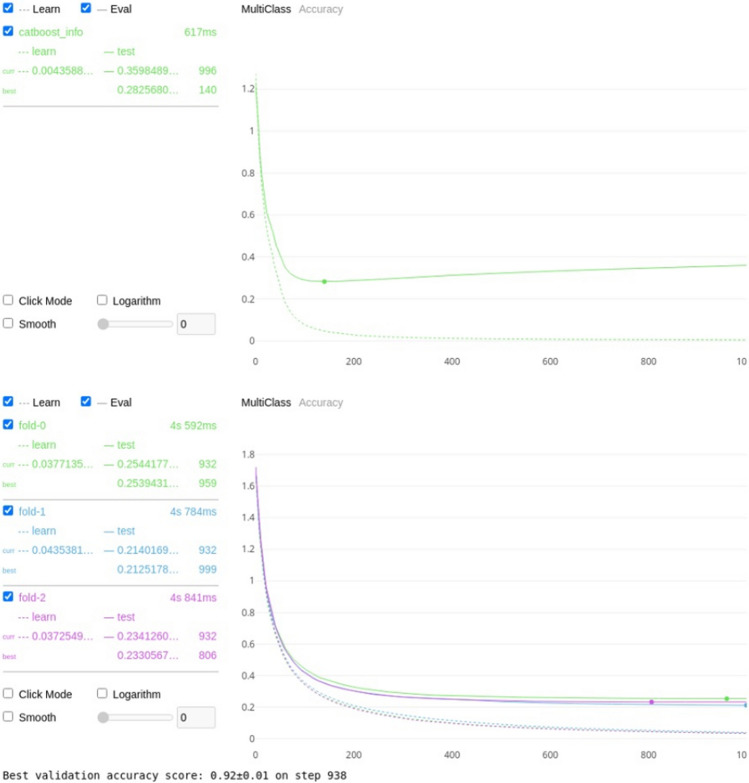
Training and validation plots for scorer 3.

Table 15 shows the results for the CatBoost classification model trained and validated against scorer 3 and Fig. 6 shows the training and validation plots to ensure no overfitting.

**Table 15:** Catboost threefold cross validation accuracy for scorer 3 for each of the 3 lameness score groups: separate degrees of lameness, scores 0 as non-lame with scores 1, 2 and 3 as lame, scores 0 and 1 as non-lame with scores of 2 and 3 as lame, scores 0, 1 and 2 as non-lame and a score of 3 as lame.

Lameness score	Threefold cross validation accuracy (%)
All	92
Lameness ≥ 1	95
Lameness ≥ 2	95
Lameness ≥ 3	99

## Discussion

The aim of this study was to develop a prototype fully automated multiple-cow lameness detection system (i.e., in multiple cows simultaneously) using a deep convolutional neural network for cattle detection and pose estimation. The algorithms developed here successfully identified lameness in dairy cows with a classification accuracy of 94 ± 0.5% using threefold cross-validation, correctly classifying the cows into the four separate degrees of lameness (0–3), as per the AHDB mobility scoring system. This contrasts with previous studies that were only able to provide a simpler binary classification of lame or non-lame cows^6,17,18^.

Currently, one of the most promising features to be analyzed automatically using visual techniques for cattle mobility scoring is back posture^10,19^, with an accuracy based on optimal thresholds exceeding 90%^19^. The proposed algorithms in this paper use back posture amongst other metrics but are entirely autonomous and do not hinder the standard milking process, whereas many of the other systems require human input^10^ and specialist equipment^6^.

**Figure 7:**
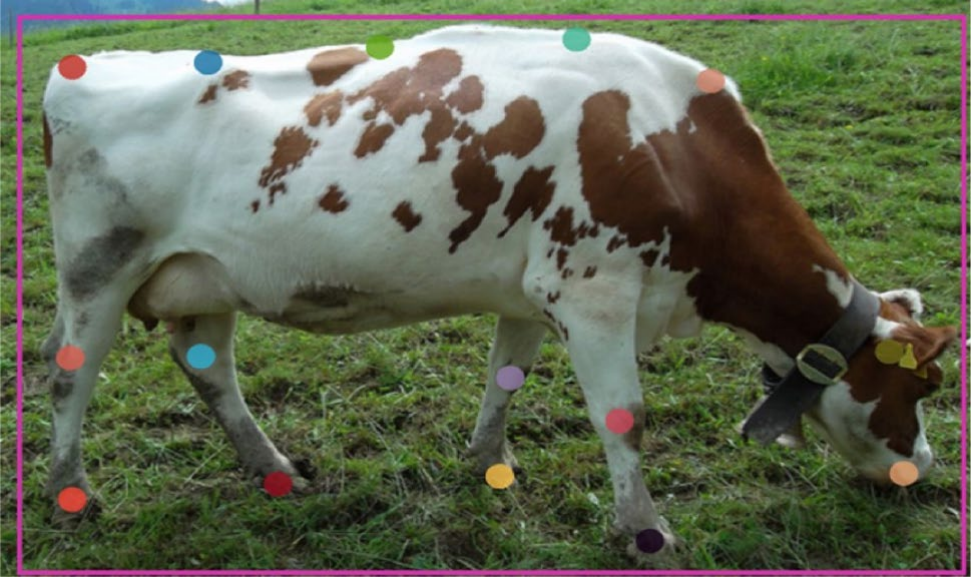
Visual description of the 15 anatomical key points, nose, head, scapula, withers, centre of the back, hook bone, tail setting, all four knees and all four hooves, used as the annotations to train the deep learning algorithm.

The Mask-RCNN pose estimation algorithm detects the essential anatomical points shown in Fig. 7 (head position, back-arching, feet and knee positions) associated with accurately identifying cattle lameness^20,21^. The algorithm can locate all the anatomical points with a high degree of accuracy. The reason for using a deep learning model over other methods like the widely used background subtraction^11,19,20,22^ is due to far greater ability to deal with image variability. The algorithm can learn what the head, nose and all the other points physically look like. Furthermore, the algorithm considers the anatomical position of each point in relation to the other anatomical points it has learnt to look for. This is more accurate than removing the foreground from the background as used by a subtraction method, which only gives a binarized image, opposed to the x, y coordinates of the feature generated by the Mask-RCNN. As the tracking algorithm was used to record the cow features as the cow moved through time (i.e., walking). This was crucial as analysing the single instance of the cow for lameness (i.e., from a single image/frame) will be a poor representation of the animal’s state, while taking all the measurements over time would allow us to understand how they are moving and get a better understanding of the overall gait and so lameness state.

**Figure 8:**
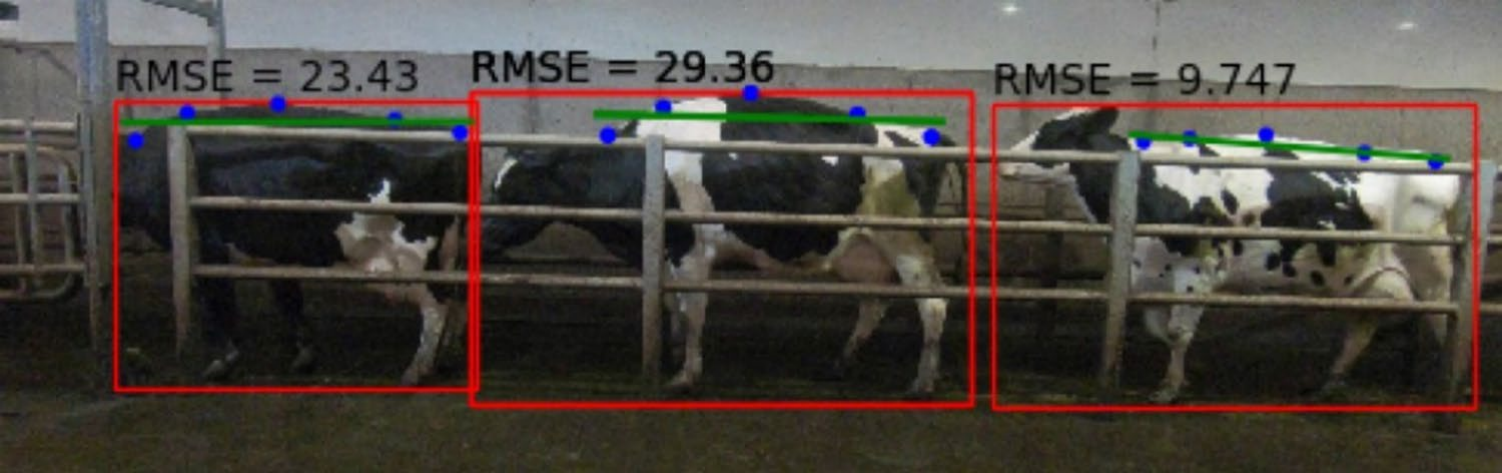
Root mean squared errors of each of the cows calculated using Eq. (1). The severely lame cow in the centre of the image shows a significantly higher score than that of the mildly lame cow (left) and sound cow (right).

The initial method developed to analyse the back posture of the cow (outlined in “Back posture analysis”) was based on the theory that as lameness increases the residuals of the back regression would also increase in magnitude, but the regression line position would remain consistent (i.e., show little deviation). Thus, the greater the root mean squared error of the residuals, the greater the mobility score. This hypothesis was shown to be correct for a single instance in time (Fig. 8). To create a more robust algorithm, each of the errors was recorded for each cow as they moved (outlined in “Tracking algorithm”). The added statistical features proved to be of considerable use in a more robust system, especially max, mean, median and standard deviation outlined in Table 1.

The mean and the median values for the back RMSE analysis shows a strong positive correlation with lameness (P < 0.001), outlined in Table 1. This increase in RMSE, as with an increase in assigned lameness, validates the algorithm as correctly picking up back arching. However, the maximum RMSE recorded as the cow moves significantly increases as the mobility score increases (P < 0.001), demonstrating that this value is a very significant feature for predicting the mobility score.

It is worth noting that there is a strong correlation of standard deviation of the back RMSE with lameness, showing that as the lameness increases the standard deviation i.e., the flexing of the back increases as the lameness score increases. This would imply that as the severely lame cow moves, it’s exhibiting considerable flexing and straightening of its spine to minimize the time spent on the affected hoof, an assumption supported by Flower and Weary^3,24^.

The second method used to analyse the back arching was dividing the back up into sections and calculating the areas above the line connecting the tail bone to the lower of the neck (outlined in “Back area analysis”) with the results outlined in Table 2. This method followed the same hypothesis that the greater the lameness of the cow, the greater the back would arch. However, this method also added further information around which area of the back was most greatly affected by the lameness. This method showed very strong correlations with lameness, with the rear of the animal holding more information than the front (outlined in Table 2). This is a crucial finding as the literature reports that the majority of causes of lameness are found to be in the hind legs of the animal^26^. Further, the results show that the back area standard deviations show a strong correlation with lameness, again implying that the greater the mobility score the greater the arching and flattening of the back as the animals move around to minimise strain on the affected area.

The rationale behind the head position analysis was that as the mobility score increased, the head movement would increase as the animal used this momentum to minimize contact time of the affected hoof with the ground, i.e., limping^26^. The initial algorithm used to understand head movement (outlined in “Head position analysis”), provided results very similar to the results obtained from the back-regression analysis, (outlined in Table 3 for the neck regression and Table 1 for back regression results respectively). This high degree of correlation potentially could have been detrimental for the final learning classification algorithm and could have damaged its ability to generalize to new data. Consequently, the neck angle algorithm was developed to look at the angle of the neck in relation to the back, (outlined in “Neck angle analysis”). This did not provide definitive results showing that there is a possible correlation between the neck angle standard deviation and the increase in lameness. However, this assumption is supported by the literature that as the cows become increasingly lame, the minimum angle of the neck tends to become more negative with the cow’s head coming closer to the ground^23^. Furthermore, the maximum angle tends to be greater, demonstrating that the head is swinging much more for a lame compared to a non-lame animal. This result confirms the hypothesis that as the mobility score increases so does the magnitude of the head movement^23^.

To ensure the greatest degree of generalization, only the most important features should be used in the final trained model, as the inclusion of nonimportant features will reduce generalization and performance. To achieve this, we tested the classification using four different permutations (outlined in “CatBoost classification algorithm”). Each required a different number of features to achieve their cumulative importance. In the first permutation (outlined in Table 5) the model requires 21 features to classify all 4 categories of mobility (0–3) with threefold cross-validation accuracy of 94 ± 0.5%. This was the most features that any of the models required. This is due to this being the hardest task and requiring the most information to discriminate between the four categories. Reviewing Table 5 it shows that the most important feature is the mean total area of the back, followed by the max and median back hip to center areas of the back further strengthening the literature^25^. A multi-variate approach, i.e., using two or more variables, was further used on this permutation to better gauge the ability to improve the model. Figure 2a shows the change in error as the features are recursively removed, with the minimum error being achieved after 4 features were removed. Figure 2b shows a focused plot of the 4 features that were removed in order to achieve the lowest error—‘neck to shoulder area skew’, ‘neck angle standard deviation’, ‘neck to shoulder area standard deviation’ and ‘neck angle kurtosis’. Although this decreased the error marginally, the overall accuracy was unaffected and this small saving in inference time for the Catboost algorithm is negligible to the overall system as most of time is used in the Mask-RCNN inference. Analysis of precision and recall for the multi-class classifier both show values close to 1 (outlined in Table 6), showing the classifier is correctly classifying examples across the multiple classes. In the second permutation (outlined in Table 7) the model is required to do a binary classification of ‘Sound’ (i.e., mobility category of 0) verses ‘Lame’ (cumulative score of mobility categories 1–3), this model required 20 features to achieve an accuracy of 98 ± 0.3%. This is only 1 less feature than required to discriminate between the 4 categories and is composed of different features. The main feature for this model is the minimum head position of the cow which is also shown as significant (outlined in Table 3) and discussed as being a feature of high importance for identifying lameness in dairy cows^23^, followed by a variation on the areas. The reason that this binary model requires a similar number of features (i.e., 20) to the classifier for the 4 categories (i.e., 21) is due to the difficulty of discriminating between lameness categories 0 (no lame) and 1 (imperfect mobility) as this difference can be considered to be only subtle. In the third permutation (outlined in Table 8) the model is required to do a binary classification of ‘Little or no lameness’ (cumulative score of mobility categories 0 and 1) versus ‘Clearly lame’ (cumulative score of mobility categories 2 and 3). This model achieved a threefold cross-validation accuracy of 100 ± 0%, with only 8 features being required. The most important feature being the mean area to the rear of the back, but all the features are orientated around the back of the cow. This shows that when the cow reaches a mobility score of 2, it is very clear by the posture of the back allowing the model to correctly classify the mobility grouping. In the final permutation (outlined in Table 9) the model is required to do a binary classification of ‘No to moderate lameness’ (cumulative score of mobility categories 0 to 2) versus ‘Severely lame’ (mobility category 3). This classifier achieved a threefold cross-validation accuracy of 97 ± 0.5% using a total of 15 features. This again shows that the back hip to the centre of the cow provides a significant amount of discriminatory information, but also shows that for mobility category 3, the head position also provides a significant amount of discriminatory information. The standard deviation is telling the classification algorithm exactly how much the cattle is moving its head, with a higher degree of head-bobbing being a known indicator of lameness^26^. With good animal husbandry, the causes of lameness are usually dealt with early and therefore the lameness classes are imbalanced with only a small number of severely lame cattle in the sample. Cohen’s kappa scores have been calculated for the four different lameness classification schemes to give an indication of the effect of the imbalance on the accuracy. For the multi-class and binary groupings of ‘sound’ [0] vs ‘lame’ [1–3] and ‘little or no lameness’ [0–1] vs ‘clearly lame’ [2–3], Cohen’s kappa ranges between 0.8162 and 0.8782 indicating the models perform very well despite imbalances in the data. The fourth model using the binary classification of ‘no to moderate lameness’ [0–2] vs ‘severely lame’ [3] returned a Cohen’s kappa of 0.502 indicating that the class imbalance, which is the most extreme of the four schemes, has a much larger effect on the accuracy and the model only performs moderately better than a random model.

To ensure our assumption of averaging the lameness scores across the scorers was valid, we trained a model for each of the scorers outlined in the methods section ‘Scorer variability CatBoost classification validation’ with the results outlined in section ‘Scorer variability CatBoost classification validation’. Through comparing the results, Scorer 1, Scorer 2 and Scorer 3 with the averaging scorer approach there is minimal difference indicating that the algorithm is finding the underlying indicators of lameness for each category and that the assumption to combine the scores through averaging was a valid decision.

Overall, this illustrates that the CatBoost model can categorise cows into the correct assigned mobility categories with a high degree of accuracy (> 94%), but that this model finds differentiating between mobility categories 0 (Not Lame) and 1 (Imperfect mobility) more difficult than then between the other categories, due to the subtle difference between these ‘not lame’ and ‘imperfect mobility’. Improving the model’s ability to differentiate between these mobility categories is the focus of this ongoing research.

This prototype system has been designed for effective implementation on farm in three phases; recording of the cow by a camera, followed by processing of video sequence by the server, followed by feedback of results on a viewing device. The camera can be placed within the parlor so that the cows can be viewed in profile (e.g., in an entry or exit race) at any distance that allows an entire cow to remain the field of view while moving a short distance (i.e., 2 × length of the cow or two strids of each foot). No additional lighting is required beyond that which is required for routine human observation of the cows. The automated analysis of lameness (from video) could be implemented using a variety of methods. Edge devices (e.g., camera, mobile or tablet), where the full processing happens on the device could be used, but at the disadvantage of performance. An on-farm server rack or computer tower (ideal) where an edge device sends the computation to the server for processing, which would achieve real-time performance (ideal), Finally, the computation could be handed off to the cloud for processing, but this could be disadvantage of performance depending on the network capability. The results of the processing could be fed back for viewing in a variety of ways; from real time viewing on an edge device (e.g., mobile phone), to local viewing like a computer as part of larger database of results. A more detailed discussion is beyond the scope of this paper, due to the variety of methods and devices that could be employed for implementation.

The prototype system described here represents a highly accurate, fully automated system that has been designed to viable for effective on-farm implementation. It offers several possible advantages over existing automated systems. It is capable dealing with multiple cows simultaneously and classifying cows according to their mobility score in accordance with a routinely used mobility scoring system (AHDB) rather being binary (i.e., lame or not lame). It tracks each individual temporally providing a better representation of each individual’s state compared to single instance sampling that is routinely used. The system is also fully autonomous and will not hinder the standard milking process.

Although, the Mask-RCNN pose estimation algorithm detects the essential anatomical points with a high degree of accuracy, this algorithm is unable to locate the feet and knees with consistent accuracy due to the low contrast between the dark feet and the dark floor. This could be improved through better lighting conditions at the farm and a more extensive training set with hard negative mining where false detections are added as negative training data. This will be the focus of future work as this tracking information is likely to be vital at pushing our accuracy greater than 94%. Future work will also include further validation on other dairy farms and diary systems to ensure that this prototype can be effectively implemented on farms and diary systems irrespective of their differences.

## Conclusions

We have successfully developed a prototype fully autonomous on-farm lameness detection system that can detect both the presence and severity of lameness to a high degree of accuracy in multiple cows. Using a standard readily available camera and convolutional neural networks (i.e., modified Mask-RCNN, SORT algorithm and CatBoost gradient boosting algorithm), the system determines lameness based upon the posture and gait of each individual by tracking key points on the back and head of each animal as they move through the camera field of view. This system has been validated against ground-truth lameness data (i.e., validated mobility scoring) and achieve an overall threefold lameness detection accuracy of 98% and a lameness severity classification accuracy (i.e., good mobility, imperfect mobility, impaired mobility, and severely impaired mobility) of 94% respectively This novel approach provides a fully automated lameness detection system, that will enable farmers to simultaneously detect early-stage lameness in multiple dairy cows on-site once it has been validated on further sites.

## Materials and methods

### Animals and management

The experimental data were collected using purely observational means (i.e., no direct manipulation) from October 2017 through April 2018 at Nafferton Farm, Newcastle University, UK. The farm has 300 lactating Holstein–Friesian cows with average milk production of 8000 kg/year. per cow. During the winter and early spring, the cows were kept indoors full time. In later spring, summer, and autumn the cows were kept outside full time and are rotated between grazing paddocks. The cows were milked twice per day in a 20-unit spaced herringbone parlor (10 places per side and 10 units per side). This farm was chosen for the initial development of the system as it represents a small to medium UK dairy enterprise which the researchers had direct access too.

The cows were video recorded to assess their locomotion when leaving one side of the milking parlor after milking to do a gait assessment^23^. On exiting the parlor, the cows walked down a single file down a 44 m walkway keeping the animals in a single file with a good viewing angle of 16 m. During this experiment, no special equipment was employed, and the farmers’ routine was not affected, to ensure the system meets its developmental aims. All animals were identified by their ear tags.

### Video acquisition

All videos were recorded using a GoPro Hero 6 at 30 frames per second (FPS) with their linear view in 2.7 K mode. The cameras were roughly set 6 m back from the cattle walkway with the walkway being lit up with two industrial site lights, as the milking parlor had poor lighting conditions. The resulting database was condensed to 25 videos, each containing 10 cows and lasting up to 2 min (n = 250). All videos were captured over a 2-week time frame.

### Gait assessment

To ensure consistency all the 25 videos were scored by three individual AHDB accredited mobility scorers using the AHDB dairy mobility scoring system, which is a 4-point score; 0 = good mobility [not lame], 1 = imperfect mobility, 2 = impaired mobility [lame], and 3 = severely impaired mobility [severely lame]^26^. The mobility scores were presented with the video sequences in a randomised order with sequences numbered 1–25. No further information was provided to the scorers. The final lameness score was assigned to the cow through taking the average of the 3 scores from the 3 separate mobility scorers and then rounding to the nearest full whole number.

### Data annotations

Each video was broken down into its constituent frames, with approximately 3600 frames per video. From each video, 1 frame in every 30 was extracted to be annotated (i.e., one frame every second). In each frame, a maximum of 3 cows could be detected in the field of view. Each cow in an image had 15 annotations (Fig. 7). To further improve the generalization of the network, another 500 images of cattle in various settings and of various types (beef and dairy cattle) were downloaded from Google using a simple JavaScript program, see Fig. 7 for one of the annotated cattle images. After combining our collected data with that of the Google image search data, the resulting database contained roughly 40,000 individual annotations.

### Pose estimation algorithm

The Mask R-CNN (mask regions with CNN features) 16 architecture was used to estimate the pose of the cows. Mask R-CNN is an extension of Region-based CNN [R-CNN] ^28^, and its faster successors^29^, which is an approach to bounding-box object detection for a variable number of objects and instances. Mask R-CNN consists of two main stages; the first stage is the Region Proposal Network [RPN] which is used to propose the bounding box object candidates. The RPN used in our Mask R-CNN implementation is the Resnet-101^30^ with the residual blocks based on the new, improved scheme proposed in^31^. The second stage extracts the features proposed by the RPN using a region of interest align [RoIAlign] to give a fixed input feature size. Then, in parallel predicts the class (in this case was either cow or background) and applies bounding-box regression and outputs a pixel coordinates for each of the 15 key-points shown in Fig. 7. Training followed that outlined in He et al.^16^ and used all the Google images to train the detector with the sampled videos (outlined in the “Data annotations”), with the error being the summation of the individual errors from the RPN class error, RPN bounding-box error, head class error, head bounding-box error, and key-point error. The mask error was removed due to the final training set not having a mask annotated as we were not using semantic segmentation. Training of the pose estimation deep learning model used all the images from the Google dataset (500 images) and images of 189 of the 250 cows recorded on the farm. This left images of 61 cows as a holdout set to perform the validation of the final system to determine mobility score, which used the outputs of the pose estimation model and performed classification on them using the CatBoost algorithm. The predictions from the final system were evaluated using threefold cross validation to give a more robust accuracy assessment. Use of the holdout set, and cross validation ensured that the end-to-end solution was validated using data which had not been used in training any part of the system previously.

### Statistical analysis

The individual cows represent the experimental unit in this study. For each of the analyses described below, Pearson’s Correlation Coefficient was used to determine the linear relationship between the generated feature datasets and the lameness score by the assessors. The correlation coefficients vary between − 1 and 1 with 0 implying there is no correlation, with the P value indicating the probability of an uncorrelated system producing datasets that have a Pearson correlation at least as extreme as the one computed from the datasets. All analysis was carried out using Python using SciPy (1.5.2).

### Back posture analysis

The root mean squared errors from a line of best fit (Eq. 1) were calculated across the five key points along the back (tail setting, hip/hook bone, the centre of the back, withers, and lower neck/scapula) detected by the pose estimation algorithm (outlined in “Pose estimation algorithm”, refer to Fig. 7). The line of best fit was found using the least square method.

1 $${\text{RMSE}} = \sqrt {\frac{1}{n}\sum\nolimits_{i = 1}^{n} {(y_{{\text{key - point}}} - y_{{{\text{line}}}} )^{2} } }$$

The RMSE gives a strong indication as to the overall posture/arching of the back, with a lower RMSE indicating a straighter, better postured back and a higher RMSE indicating greater curvature in the back and a worse posture (Fig. 8).

### Back area analysis

**Figure 9:**
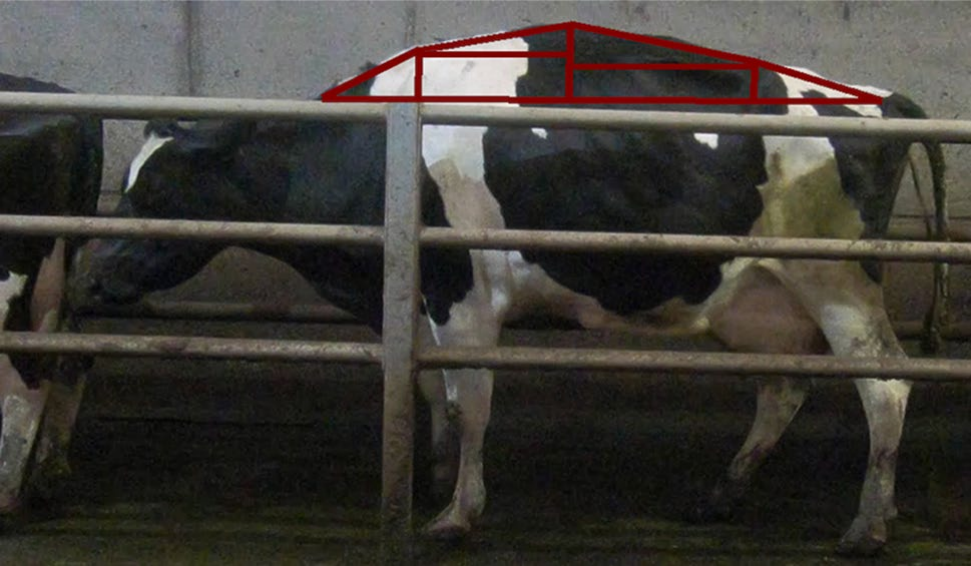
Visualization of how the area of the back is calculated by breaking it up into small sections and then summing the area of each of the sections.

Further analysis into the back posture was undertaken by using the five key points on the back to calculate the approximate area of the back. This was achieved by drawing a baseline connecting the tail setting to the lower neck/scapula and a series of toplines which join the key-points. A perpendicular line is drawn to connect each of the three intermediate key-points (withers, the centre of the back, hook bone) to the baseline, and lines parallel to the baseline are drawn between the central perpendicular line and the second and fourth key-points to allow the back area to be calculated through the summation of four triangular and two rectangular sections that provide a close estimation of the back arching area (Fig. 9).

### Head position analysis

AHDB dairy mobility scoring system identifies that the head position of the cow is another good indicator of the lameness of the cow^27^. To understand how the head is moving in relation to the rest of the body, the line of best fit (outlined in “Back posture analysis”) was continued past the head and the squared distance from nose and head key-points to the regression line was calculated. As the position signified by the sign relative to the body is essential as it indicated whether the head was held above or below the regression line, the output is multiplied by − 1 if the value was negative before the square, see Eq. (2).

2 $${\text{POS}} = \left( {{\text{y}}_{{{\text{line}}}} - {\text{y}}_{{\text{key - point}}} } \right)^{{2}} * - {1},\left( {{\text{y}}_{{{\text{line}}}} < {\text{y}}_{{\text{key - point}}} } \right)$$

### Neck angle analysis

Further analysis of the head position was carried out by analysing the angle of the neck in relation to the back. This was achieved by firstly calculating the gradient of the line from the front shoulders to the lower neck/scapula, then calculating the gradient of the line connecting the lower neck to the head. Using these gradients, the angle between them is calculated.

3 $$NeckAngle = \tan^{ - 1} \frac{{m_{{_{1} }} - m_{2} }}{{a + m_{{_{1} }} m_{2} }}$$

### Tracking algorithm

**Figure 10:**
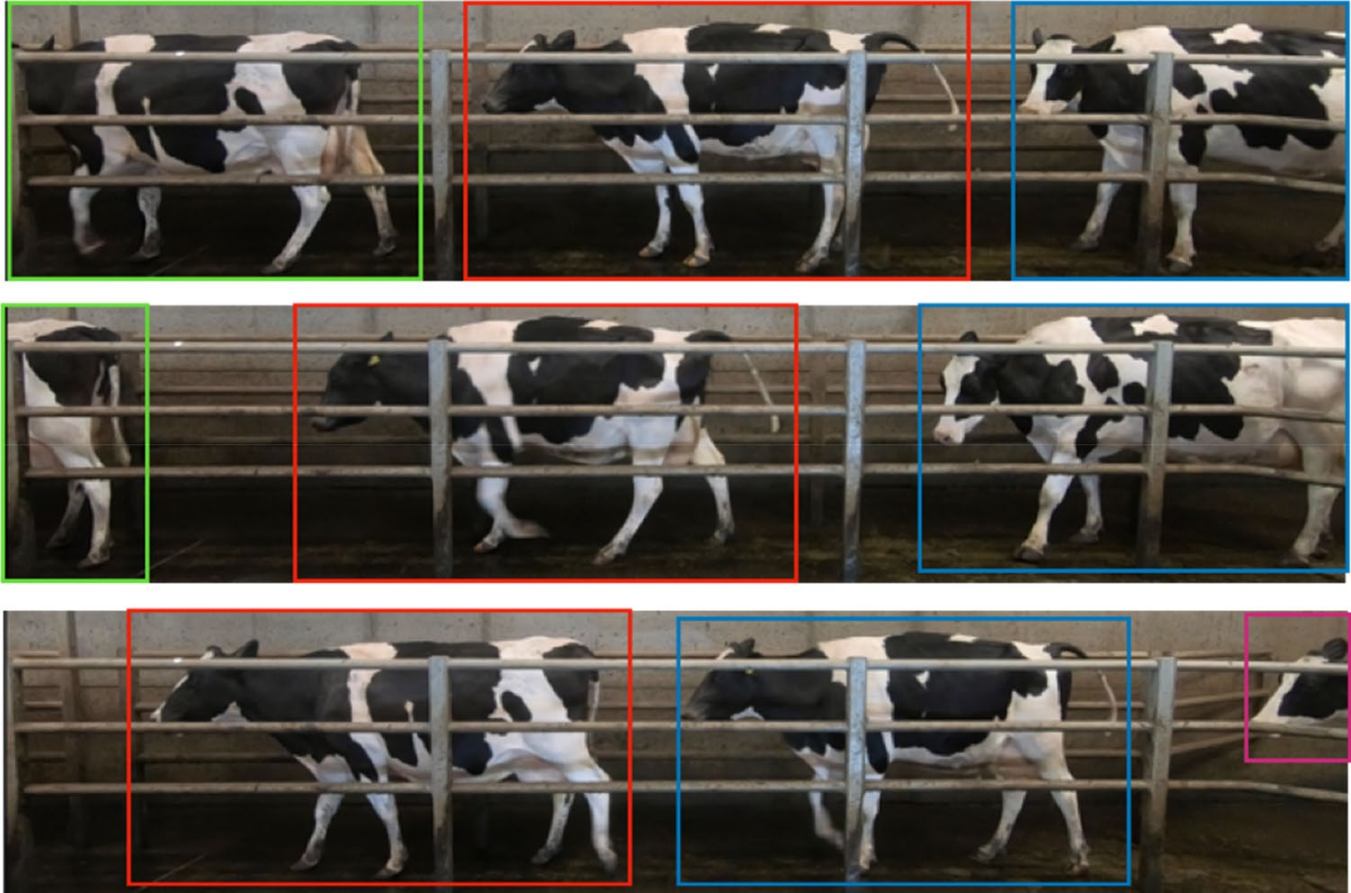
Visualization of the tracking algorithm. The top image shows three cows in the first frame, each marked with a different colour bounding box. The second frame down shows the cows 1 s later with the tracking algorithm associating the correct cows with the coloured bounding box. The bottom frame again shows the cows 1 s after the second frame.

Up until this point, all the analysis is based on a single still image and has no information on how the cows move through time. To monitor the cows through time, the Simple Online and Realtime Tracking [SORT] algorithm^32^ was selected and utilized. This algorithm was selected due to the heavy constraints placed on the cows by the walkway barriers, i.e., the cows were in single file and could not pass each other. Using SORT, the pose of the cows was monitored over time allowing us to obtain information on back regression, back area, neck regression and the neck angle, e.g., min value, max value, mean, median, standard deviation, kurtosis and the skew as the cow moved. Figure 10 shows a visualization of the tracking algorithm.

### CatBoost classification algorithm

CatBoost is a supervised machine learning library for gradient boosting on decision trees^33^. The CatBoost algorithm is designed for heterogeneous data where the columns are a feature or predictor, such as the area of the back, the angle of the neck and each row is some observation of the cow. For each observation, there is a label indicating the mobility score between 0 and 3^27^. The mobility score is the target output which needs to be predicted by the network based on the description of observations in the form of a vector of the features. The method works by iteratively learning weak classifiers and then adding them together, to form a strong classifier^34^. After a weak learner is added, the data is then re-weighted so that incorrectly classified examples gain weight, and the correctly classified examples lose weight. By doing this, the future weak learners are forced to concentrate more on the misclassified samples. Many of the trees are added together using a gradient descent procedure^35^ to minimize the error when adding trees, with the error function being any differential function.

To test the accuracy of the trained CatBoost model, threefold cross-validation was used to average the test results^36^. K-fold cross-validation was done with a hold-out percentage of 20% to provide a more significant assessment of the CatBoost algorithm on our dataset with K-fold cross-validation essentially using all the data for training and all the data for testing. For each of the three folds, using a 20% hold-out (i.e., test data), left a training set of 80% of the data. In the first fold, the model trained on this 80% with the accuracy tested on hold-out 20% and then stored. This model was then discarded, the data then reshuffled and new training (80%) and hold-out sets (20%) created. In the second fold, a new model was then trained new 80% of the data, and the accuracy tested on new hold-out 20% and then stored. This was repeated one final time for the third fold. With the three foldout accuracies being averaged for the accuracy of the system, meaning no test data was present in the training set when each model was created. Four different models were created with each model being evaluated using threefold validation. The first model was developed to correctly classifying each cow into its assigned mobility category (0, 1, 2 or 3). The second model was developed to correctly classifying each cow as either ‘Sound’ (i.e., assigned mobility score of 0) or ‘Lame’ (assigned mobility scores of 1, 2 and 3 combined^27^). The third model was developed to correctly classifying each cow as either exhibiting ‘Little or no lameness’ (i.e., assigned mobility scores of 0 and 1 combined) or ‘Clearly lame’ (assigned mobility scores of 2 and 3 combined). The final model was developed to correctly classifying each cow as either ‘Very obviously lame; (assigned mobility score of 3) or ‘Not obviously lame’ (i.e., assigned mobility scores of 0, 1 and 2 combined). Testing accuracy in this manner allows the weaknesses (i.e., differentiating between lameness categories) of the CatBoost model to be identified ensuring the greatest degree of generalization and performance, as only the most important features should be used in the final trained model. The removal of features that have not inputted to the final solution reduces noise in the data due to these features. In addition, removing highly correlated features also avoids skewing the results. To test for further improvements on the accuracy of the model, a recursive feature elimination algorithm was used to test for multi-variate feature interpretation on the Catboost model. The recursive feature elimination algorithm works through fitting a model and removes the weakest feature (or features) until a set number of features is reached. To further assess the success of our Catboost classifier, Cohen’s kappa coefficient, precision and recall were calculated. Precision is the measure of the results relevancy, whilst the recall is the measure of how many truly relevant results are returned. Both were calculated from the confusion matrix. This was deemed necessary as the dataset was not perfectly balanced.

### Scorer variability CatBoost classification validation

To validate whether the decision to average the 3 lameness scorer scores into a single score and use that as a target variable to train our models against, we trained a model against each of the individual lameness scorers and validated the performance with threefold cross validation. This will indicate whether the model is generalising to the underlying indicators of lameness.

### Ethical approval

This study received ethical approval (ID689) from the Animal Welfare and Ethics Review Board (AWERB) of Newcastle University. This was a purely observational study on animals undergoing routine husbandry procedures, which were carried out in accordance with the UK Animal Welfare Act 2006. No further approval under the UK Animals (Scientific Procedures) Act 1986 was required as the animals were simply observed while undergoing routine husbandry without manipulation for experimental purposes. The manuscript was compiled in accordance with the ARRIVE guidelines.

## Data Availability

The datasets generated during and/or analysed during the current study are not publicly available due to ongoing development of the algorithms but are available from the corresponding author on reasonable request.
